# The spatial biology of transcription and translation in rapidly growing *Escherichia coli*

**DOI:** 10.3389/fmicb.2015.00636

**Published:** 2015-07-02

**Authors:** Somenath Bakshi, Heejun Choi, James C. Weisshaar

**Affiliations:** Department of Chemistry and Molecular Biophysics Program, University of Wisconsin-Madison, MadisonWI, USA

**Keywords:** single-molecule tracking live cell, *E. coli*, ribosomes, RNA polymerase, nucleoid structure, DNA-ribosome spatial segregation

## Abstract

Single-molecule fluorescence provides high resolution spatial distributions of ribosomes and RNA polymerase (RNAP) in live, rapidly growing *Escherichia coli*. Ribosomes are more strongly segregated from the nucleoids (chromosomal DNA) than previous widefield fluorescence studies suggested. While most transcription may be co-translational, the evidence indicates that most translation occurs on free mRNA copies that have diffused from the nucleoids to a ribosome-rich region. Analysis of time-resolved images of the nucleoid spatial distribution after treatment with the transcription-halting drug rifampicin and the translation-halting drug chloramphenicol shows that both drugs cause nucleoid contraction on the 0–3 min timescale. This is consistent with the transertion hypothesis. We suggest that the longer-term (20–30 min) nucleoid expansion after Rif treatment arises from conversion of 70S-polysomes to 30S and 50S subunits, which readily penetrate the nucleoids. Monte Carlo simulations of a polymer bead model built to mimic the chromosomal DNA and ribosomes (either 70S-polysomes or 30S and 50S subunits) explain spatial segregation or mixing of ribosomes and nucleoids in terms of excluded volume and entropic effects alone. A comprehensive model of the transcription-translation-transertion *system* incorporates this new information about the spatial organization of the *E. coli* cytoplasm. We propose that transertion, which radially expands the nucleoids, is essential for recycling of 30S and 50S subunits from ribosome-rich regions back into the nucleoids. There they initiate co-transcriptional translation, which is an important mechanism for maintaining RNAP forward progress and protecting the nascent mRNA chain. Segregation of 70S-polysomes from the nucleoid may facilitate rapid growth by shortening the search time for ribosomes to find free mRNA concentrated outside the nucleoid and the search time for RNAP concentrated within the nucleoid to find transcription initiation sites.

## Introduction

Super-resolution fluorescence methods (Betzig et al., 2006; Hess et al., 2006; Rust et al., 2006) enable detailed exploration of the ways in which the central dogma of molecular biology plays out in *Escherichia coli*. “Photoactivation-localization microscopy” (PALM) can locate and track 1000s of copies of single, specific proteins *in live cells* with ∼30 nm spatial resolution and low-ms time resolution. This has enabled detailed, quantitative studies of how ribosomes (Bakshi et al., 2012), chromosomal DNA (Wang et al., 2011), and RNA polymerase (RNAP; Bakshi et al., 2012, 2013; Endesfelder et al., 2013) are distributed in space and move in time within the cytoplasm of single cells. In addition, time-resolved widefield fluorescence imaging after drug treatment has provided new insight into the ways in which transcription and translation determine the internal organization of cytoplasm (Bakshi et al., 2014a). Transcription, translation, and the spatial organization of *E. coli* cytoplasm act as a coupled biochemical–biophysical system. Here, we describe the delicate balance of forces that enables the system to drive rapid cell growth.

**FIGURE 1:**
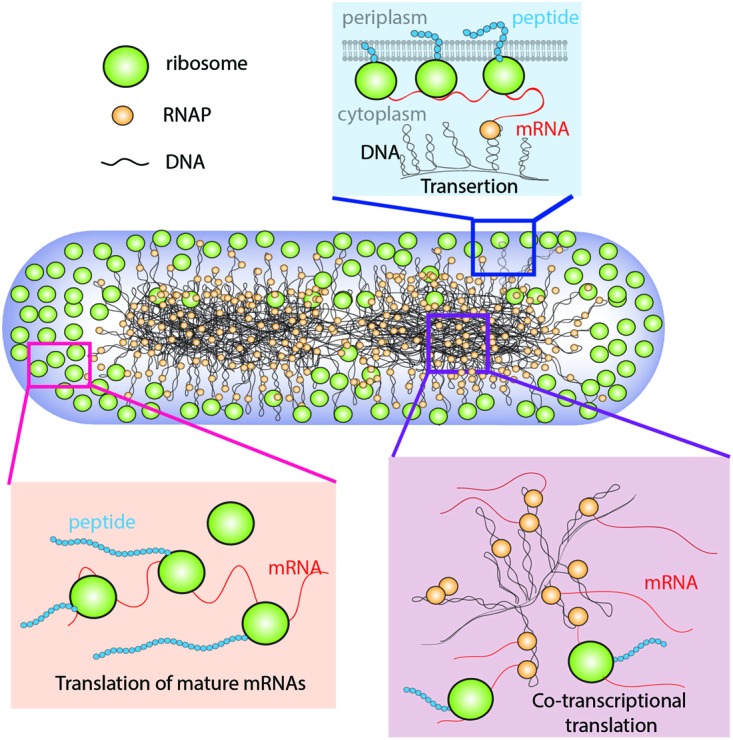
**Schematic of the coupled transcription–translation–transertion system in rapidly growing *Escherichia coli*.** Adapted from Bakshi et al. (2014a).

In *E. coli*, the chromosomal DNA occupies the region of space called the *nucleoids* (Kellenberger, 1991). During rapid growth, ribosomes are concentrated outside the nucleoids in *ribosome-rich regions* comprising the two polar end-caps, the space between nucleoid lobes, and the thin region proximal to the cytoplasmic membrane (**Figure 1**; Bakshi et al., 2012). The spatial extent of the nucleoids evidently arises from a balance of compacting and expanding forces (Woldringh et al., 1995; Zimmerman, 2006). Likely compacting forces include depletion-attraction of DNA arising from macromolecular crowding by myriad small proteins (Zimmerman and Murphy, 1996); conformational entropy of the confined DNA polymer, which causes the polymer to avoid walls (Mondal et al., 2011; Bakshi et al., 2014a); inter-strand coupling by DNA binding proteins such as H-NS (Dame, 2005; Wang et al., 2011); bending of DNA by IHF (Dame, 2005); and net supercoiling of the DNA by Gyrase and Topoisomerase I (Woldringh et al., 1995). The hypothesized primary expanding force is “*transertion*,” which is the simultaneous co-transcriptional translation *and insertion* of membrane proteins via the translocon machinery (Woldringh, 2002). Transertion implies the existence of DNA-RNAP-mRNA-ribosome-polypeptide-membrane *“transertion chains”* directly linking DNA to the membrane (**Figure 1**). A sufficient number of these chains would radially expand the overall nucleoid. The main evidence for transertion had been the dramatic contraction of the nucleoids on treatment with translation-halting drugs such as chloramphenicol (van Helvoort et al., 1996; Zimmerman, 2002). Treatment with transcription-halting drugs such as rifampicin should have the same effect, but this was not observed on the 30-min timescale studied (Fishov and Woldringh, 1999; Cabrera et al., 2009). Our recent time-dependent imaging study discovered nucleoid contraction on a 3-min timescale after rifampicin treatment, placing the transertion hypothesis on solid footing (Bakshi et al., 2014a).

In rapidly growing *E. coli*, only 10–15% of ribosomal subunits lie within the nucleoids (Bakshi et al., 2012). Single-30S subunit tracking studies suggest that most 30S subunits in the ribosome-rich regions are engaged as slowly diffusing 70S-polysomes. The majority of the translation events are presumably carried out on mature, freely diffusing mRNAs within the ribosome-rich regions. While *co-transcriptional* translation (coupled transcription and translation; Miller et al., 1970) is essential for protecting nascent mRNA and ensuring efficient transcription (Burmann et al., 2010, 2012; Proshkin et al., 2010; Svetlov and Nudler, 2012; McGary and Nudler, 2013), it is apparently not the primary means of protein production. The 70S-polysome diffusion coefficient *D_70S-poly_* ∼ 0.02 μm^2^ s^-1^ is large enough to enable nascent mRNA to diffusively find a ribosome-rich region in ∼1 s (Bakshi et al., 2012), a short period compared with the ∼6–7 min lifetime of mRNA against degradation (Bernstein et al., 2002).

A number of earlier studies used physical models to explain the formation of compact nucleoids that occupy only a fraction of the entire bacterial cytoplasm. A statistical mechanical model used the osmotic effects of myriad small proteins to explain the apparent phase separation (Odijk, 1998). A recent experimental study of free bacterial nucleoids confined in a microfluidic channel showed that crowding by added polyethylene glycine chains (PEG) led to reversible, first-order “coil-to-globule” collapse of the nucleoids (Pelletier et al., 2012). This study augmented the earlier statistical model to include the entropic spring nature of the nucleoids. A very recent coarse-grained simulation showed that small, spherical crowding agents can induce compaction of a DNA polymer, modeled as a freely jointed chain (Shendruk et al., 2015).

We have found that a simple physical model using Monte Carlo simulations of DNA and ribosome spatial distributions in a confining cytoplasmic space enhances our understanding of the observed ribosome-nucleoid segregation (Mondal et al., 2011). In the model, entropic and excluded volume effects cause strong segregation of the unperturbed nucleoid from 70S-polysomes. The biochemical state of ribosomes (70S-polysomes vs. free 30S and 50S subunits) plays an essential role in ribosome-nucleoid segregation. When the model 70S-polysomes are converted to 30S and 50S subunits, the components mix quite thoroughly with the DNA and the nucleoid expands (Bakshi et al., 2014a). This suggests that the fraction of 70S-polysomes vs. 30S and 50S subunits strongly affects the relative compactness of the nucleoid.

Based on these new experimental and computational results, we are developing a comprehensive model of the spatial organization within the *E. coli* cytoplasm and how it may work to optimize cell growth. Our present view encompasses a variety of inter-related phenomena: (1) In rapidly growing cells, *most translation occurs in the ribosome-rich regions*, not within the nucleoids. (2) Yet there is direct evidence of co-transcriptional translation (Miller et al., 1970), and this is important for protecting nascent mRNA from degradation and for efficient transcription. *Evidently most or all transcription is co-translational, but only ∼10–15% of translation is co-transcriptional*. (3) Therefore 30S and 50S subunits must be able to penetrate the nucleoid where they initiate co-transcriptional translation (Sanamrad et al., 2014). This picture implies a *circulation* of 30S and 50S subunits between the nucleoids and the ribosome-rich regions. (4) Transertion expands the nucleoids beyond their relaxed, highly compacted state (Woldringh, 2002). The evidence suggests that transertion plays an essential role, enabling 30S and 50S subunits to move into the nucleoids where they initiate co-transcriptional translation and form nascent 70S-polysomes.

In this view, the transcription and translation machinery and the spatial organization of the cytoplasm act as a coupled biochemical–biophysical system. Effective compartmentalization of the cytoplasm into ribosome-rich regions and DNA- and RNAP-rich nucleoids may enhance the efficiency of protein synthesis and the utilization of RNAP. Segregation of 70S-polysomes from the nucleoid may facilitate rapid growth by minimizing the search time for ribosomes to find the free mRNA concentrated outside the nucleoid and for RNAP concentrated within the nucleoid to find transcription initiation sites. This review describes the evidence behind the comprehensive picture, briefly compares *E. coli* with *Caulobacter crescentus*, and contrasts our “translation-centric” view of nucleoid morphology with a “transcription-centric” view (Jin et al., 2013) presented in another chapter of this volume.

## Super-Resolution Imaging of Single Protein Copies in Live Bacterial Cells

### Overview

**FIGURE 2:**
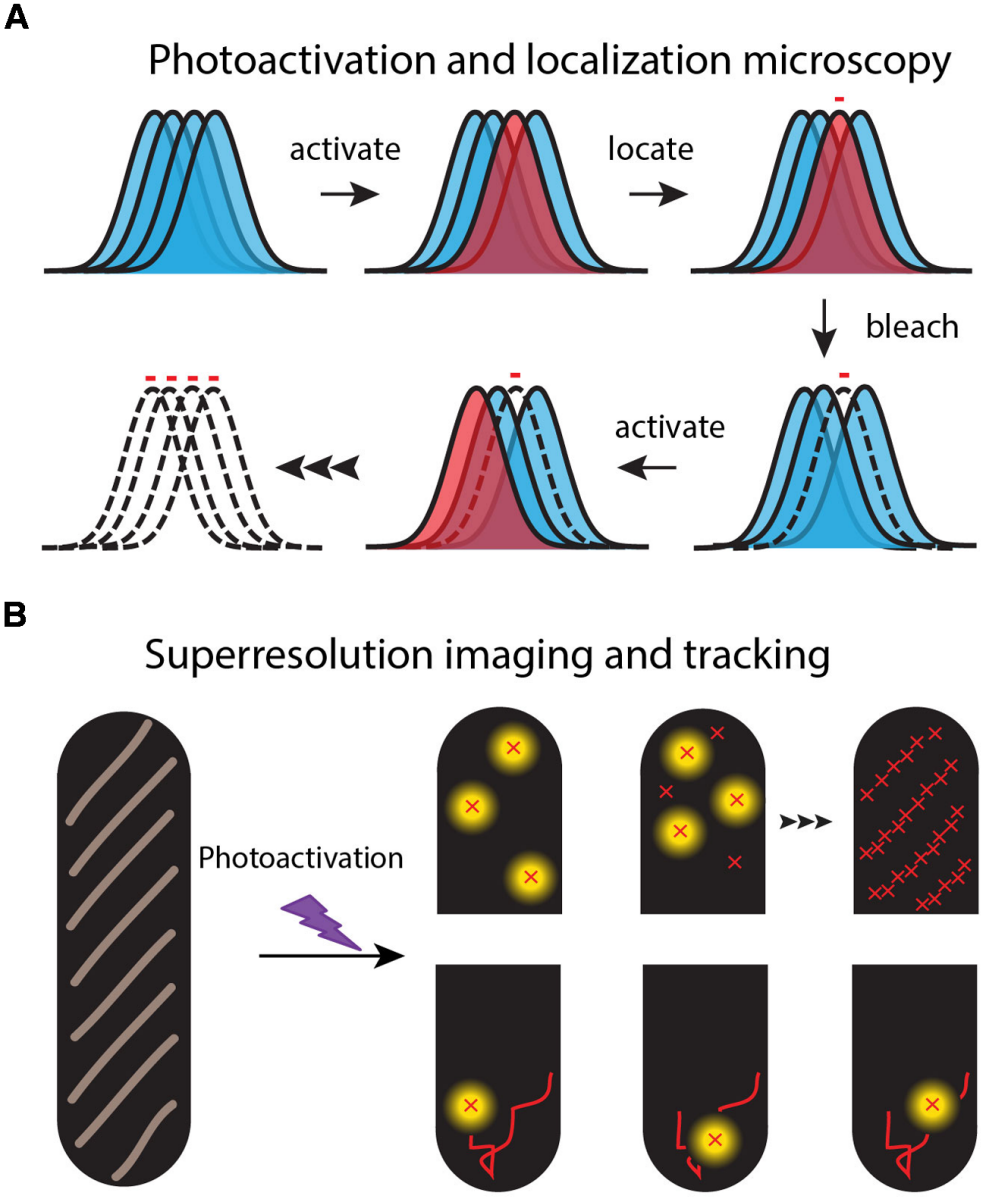
**Schematic of the super-resolution imaging method. (A)** Each fluorescent molecule makes a diffraction-limited, essentially Gaussian image on the camera. A dense set of normal labels (blue images) makes overlapping images. In each imaging cycle, a sparse set of labels is photoactivated (red image) and localized from the well-isolated, single-molecule images. **(B)** An image of the spatial distribution is built up one molecule at a time over 100s or 1000s of optical cycles (upper “half-cells”). Successive images of the same molecule form a diffusive trajectory (lower “half-cells”).

Super-resolution fluorescence microscopy of specific proteins in live cells (**Figure 2**) was enabled by three key technical advances. First, genetic manipulations enable replacement of the gene for the target protein by a gene that appends a fluorescent protein to the target. This “GFP revolution” enables imaging of a specific target protein in a live cell (Zhang et al., 2002). But caution is needed. The fluorescent protein tags may affect the function, aggregation state, spatial distribution, or movement of the target protein (Landgraf et al., 2012).

Second, on a dark background a single fluorescent molecule can produce a punctal, high signal-to-noise image on a sensitive EMCCD camera (Vrljic et al., 2007). However, the full width at half maximum height (FWHM) of this image is broadened to ∼λ/2 = 260 nm for detection of green light with wavelength λ = 520 nm. This defines the diffraction limit of light for an optical microscope. Nevertheless, a well-isolated, slowly moving single fluorophore can be located and tracked far more accurately than the diffraction-limited width of its image. In a 1 μm diameter × 3 μm long *E. coli* cell, the localization accuracy for fluorescent proteins is photon- and background-limited to ∼30 nm in each of the two projected dimensions (Bakshi et al., 2011).

Third, the super-resolution fluorescence methods use “photoswitchable” fluorescent proteins as the labels to overcome the problem of spatial overlap of the myriad images of high copy number fluorescent proteins in small cells (**Figure 2**). For example, PALM and its variations enable sub-diffraction-limit imaging of the ∼50,000 labeled 30S-mEos2 ribosomal subunits, essentially by imaging them one or a few copies at a time (Betzig et al., 2006; Rust et al., 2006; Gould et al., 2008). In a widefield fluorescence image, all the 30S images would overlap in space, severely limiting the precision of spatial information (**Figure 2**). Following excitation at shorter wavelengths (typically near 405 nm), photoswitchable proteins undergo photochemistry that shifts their absorption and emission spectra toward longer wavelengths. For the photoswitchable label mEos2, PALM uses weak excitation at 405 nm to create a sparse set of new fluorophores that emit at 584 nm. In each PALM cycle, the few photoswitched copies are located and tracked by excitation with a second probe laser at 561 nm (where the unswitched copies do not absorb). The photoswitched copies bleach all too quickly, after which a new sparse set is photoswitched and tracked. Over several-minutes, literally 1000s of short, 5–15 step trajectories with ∼30 nm localization accuracy can be acquired. The stored individual locations are used to reconstruct a PALM image of the two-dimensional spatial distribution of the target protein, averaged over a typical 1–2 min of data acquisition.

The diffusive behavior of each individual copy then provides clues to its biochemical state (Lippincott-Schwartz and Patterson, 2009). For ribosomes, 70S-polysomes diffuse somewhat more slowly than 30S subunits searching for a translation initiation site (Bakshi et al., 2012). Those RNAP copies that are searching for a transcription initiation site diffuse much more rapidly than copies engaged in transcription and thus bound to the DNA polymer (Bakshi et al., 2013). Thus PALM provides not only much more precise spatial distributions than widefield microscopy, but also new information about where specific biochemical processes are carried out within the bacterial cell and the fraction of proteins engaged in each process.

### Cell Growth and Preparation

Our preferred *E. coli* strain VH1000 is a modification of MG1655 with the PyrE defect repaired. VH1000 grown in EZRDM (“EZ rich, defined medium”; Neidhardt et al., 1974), MBM (MOPS-buffered minimal medium) using glucose as carbon source, or MBM with glycerol, provides access to a wide range of doubling time (30, 60, and 120 min, respectively, at 37°C). Most of the experiments described here were carried out in EZRDM at 30°C.

We maintain cells at well-defined, constant levels of nutrition and aeration throughout the imaging experiments (Bakshi et al., 2011, 2012). Cells are harvested from exponential growth and plated on a polylysine coated coverslip that forms the base of a microfluidics device providing a continuous flow of fresh, aerated growth medium during imaging. This enables us to study 50–100 cells simultaneously. Length vs. time measurements of single cells shows that they grow at the same rate as in bulk medium to within 5%; evidently there are no harmful effects of the polylysine surface. Visible light can be toxic (Bakshi et al., 2012). It is essential to minimize total photon dosage at 514 or 561 nm. We always ensure that the laser exposure does not alter the property being measured.

### Labeling Strategies

The use of λ-Red mediated recombination (Datsenko and Wanner, 2000) to replace wild-type genes on the chromosome with genes for mEos2-labeled proteins has become routine. P1 transduction then transfers the new genes back to the parent strain to eliminate unwanted mutations. Our VH1000 strains expressing the ribosomal protein S2 labeled by mEos2 or the RNAP subunit β′ labeled with mEos2 grow normally. The long time required for mEos2 to achieve its fluorescent state (∼130 min) compared to the assembly time of ribosome/RNAP core enzymes (a few min) ensures that we are almost always tracking assembled 30S ribosomal subunits (Bakshi et al., 2012) or complete RNAP enzymes (Bakshi et al., 2013). As a test for possible adverse effects of the labels (Landgraf et al., 2012), we showed that for both β′ and S2, the mEos2 and yGFP labeling schemes produce indistinguishable spatial distributions and diffusive properties.

To image the overall spatial distribution of DNA in growing cells, we strongly prefer the non-perturbative stain SYTOX Orange over the more standard DAPI imaging (Bakshi et al., 2014a,b). DAPI staining plus UV light perturbs growth rate and nucleoid morphology. SYTOX Orange staining enables normal growth and widefield imaging over 100s of camera frames.

### Single-Molecule Localization and Tracking Methodology

The mEos2 constructs yield trajectories of mean length 10 frames and enable control of the time between frames. For each 1000 trajectories, we obtain ∼150 trajectories of length >18 frames. We spatially filter the images and fit single-molecule locations using a centroid algorithm (Wang et al., 2010; Bakshi et al., 2011, 2012). Images of cells are rotated so that the long axis is *x* and the short, transverse axis is *y*. By plotting a point at the (*x, y*) coordinates of each centroid, we produce a high-resolution, two-dimensional (2D) projection of the 3D spatial distribution, averaged over the several-minute acquisition period.

A sequence of locations for a particular particle forms a diffusive trajectory. We analyze single-molecule diffusion data in a variety of ways. Plots of mean-square displacement vs. lag time τ, *MSD*(τ), are obtained as a running average over each trajectory and over all molecules. To test for sub-diffusion effects due to tethering or caging, *MSD*(τ) is compared with Monte Carlo random walk (free diffusion) model calculations within an appropriate confinement volume. To test for heterogeneity of diffusion, we compute histograms of single-molecule diffusion coefficients, estimated as *D_i_* = msd_*i*_(τ)/4τ, where msd*_i_*(τ) is the single-molecule mean-square displacement averaged over *one trajectory*. The aspect ratio of trajectories can also help distinguish sub-diffusion from free diffusion of single-molecules (Bakshi et al., 2013).

## Ribosome-Nucleoid Segregation in Rapid, Exponential Growth

### Background

Miller et al. (1970), in their EM study of the contents of the *E. coli* cytoplasm observed long DNA strands to which chains of ribosomes (“70S-polysomes”) were attached, direct evidence of translation coupled to transcription (“co-transcriptional translation”). That study inferred that *all* bacterial protein synthesis was co-transcriptional. The total amount of mRNA per cell and the ribosome copy number confirm the importance of polysomes and suggest their typical length to be about ten 70S per mRNA. Co-transcriptional translation evidently assists optimal cell growth. It helps protect nascent mRNA against early termination by Rho and against premature degradation by ribonucleases (Proshkin et al., 2010; McGary and Nudler, 2013). In addition, specific proteins bind simultaneously to both RNAP and the lead ribosome in a polysome chain (Burmann et al., 2012; Svetlov and Nudler, 2012). The translating lead ribosome helps to prevent undesirable RNAP backtracking (Burmann et al., 2010).

However, evidence from EM studies of sections of fixed *E. coli* cells (Kellenberger, 1991) and from widefield immuno-staining of whole, fixed cells (Azam et al., 2000) argued against a model in which all translation is co-transcriptional. The images showed a strong tendency of ribosomes to avoid the nucleoids. Early widefield fluorescence studies in fixed *Bacillus subtilis* co-imaged DNA and ribosomes and again demonstrated strong segregation of ribosomes from DNA (Lewis et al., 2000).

**FIGURE 3:**
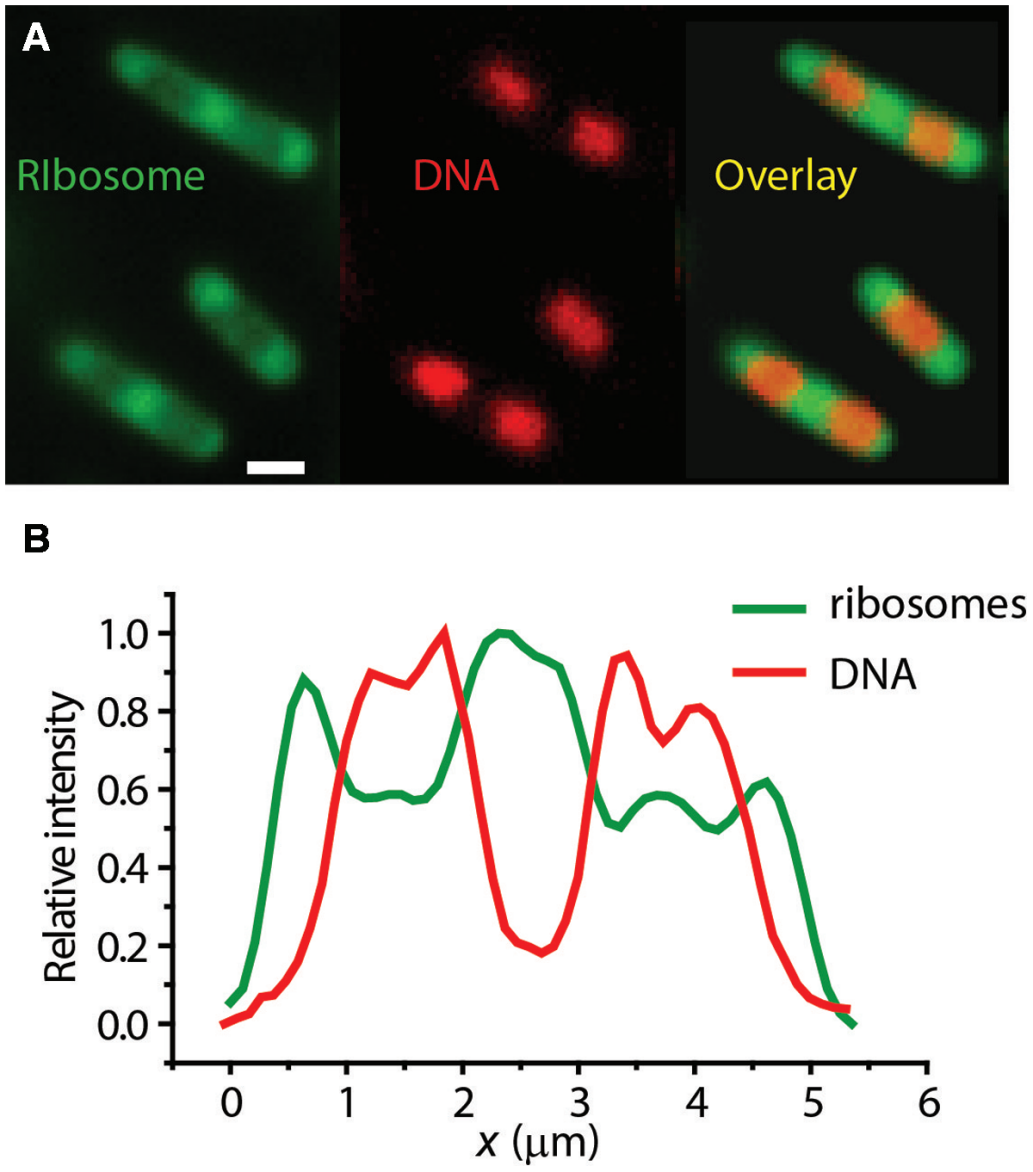
**(A)** Widefield images of ribosomes (S2-YFP labeling) and DNA (DRAQ5 staining) in single *E. coli* cells. **(B)** Axial linescans of ribosome and DNA intensity vs. the long-axis coordinate *x*. Anti-correlation is evident. Adapted from Bakshi et al. (2012).

Our own widefield images of live *E. coli* with DNA stained with DRAQ5 and ribsosomes labeled with S2-YFP show clear anti-correlation in the axial spatial distributions of the two species (**Figure 3**). The “peak-to-valley” ratio of the ribosomes is about 1.5:1. Intriguingly, in the slow-growing species *C. crescentus*, ribosomes and DNA appear to be much more thoroughly mixed (Llopis et al., 2010).

### Superresolution Imaging of RNAP and Ribosomes

**FIGURE 4:**
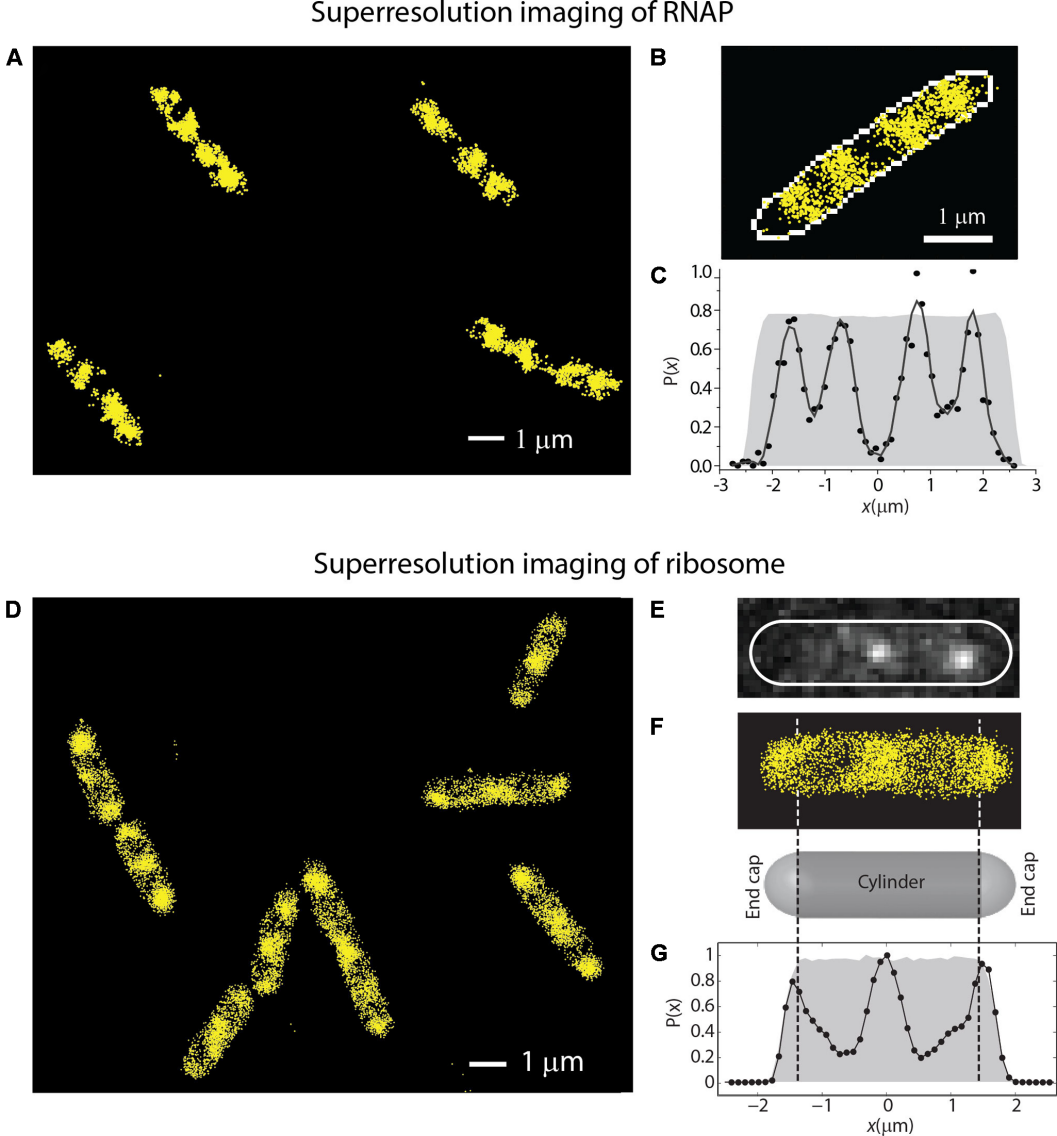
**(A–C)** Super-resolution imaging of the spatial distributions of RNA polymerase (β′-YFP labeling) in live *E. coli*. **(C)** Shows a histogram of locations projected onto the long axis of the cell in **(B)**. **(D–G)** Super-resolution imaging of the spatial distributions of ribosomes (30S-YFP labeling) in live *E. coli*. **(E)** Shows two single copies imaged in one camera frame. **(G)** Shows an axial histogram of locations for the cell shown in **(F)**. The gray regions in **(C,G)** are simulated histograms for a uniformly filled spherocylinder matching the length and radius of the single cell. Adapted from Bakshi et al. (2012).

In Bakshi et al. (2012), we reported super-resolution images of RNAP (β′-yGFP, **Figures 4A–C**) and ribosome (30S-YFP, **Figures 4D–G**) spatial distributions. RNAP spends almost all of its time bound to DNA, either specifically or non-specifically. The experiments revealed a much greater degree of RNAP/ribosome spatial segregation than suggested by the earlier widefield work.

**FIGURE 5:**
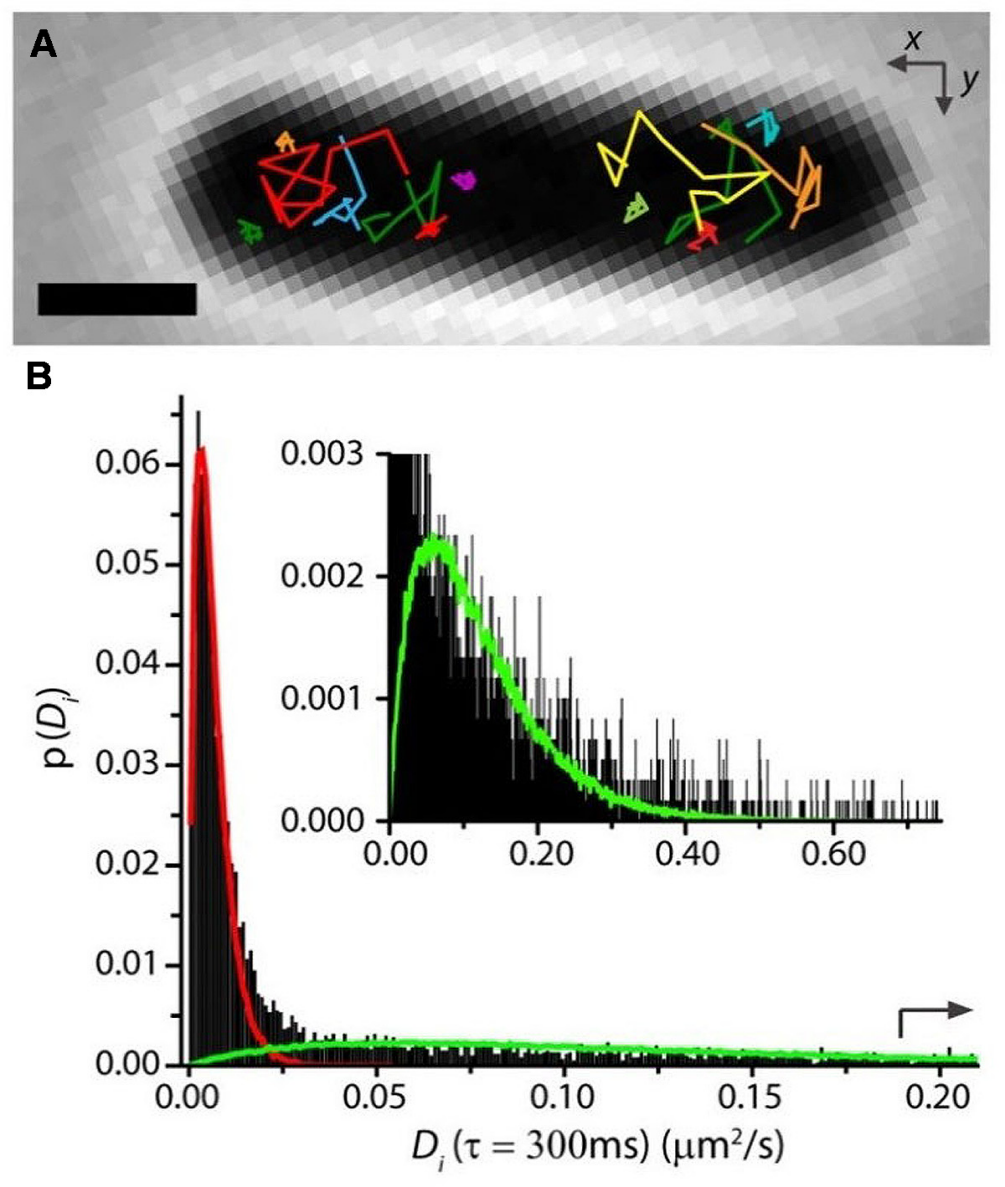
**(A)** Single-RNAP diffusive trajectories in live *E. coli* growing in EZRDM at 37°C. Labeling was β′-mEos2. Note two types of trajectory. **(B)** Distribution of estimated diffusion coefficients *D_i_* from single-RNAP trajectories. Fast and slow sub-populations are evident. Red and green curves are model distributions for the two components. Adapted from Bakshi et al. (2013).

In our study of RNAP motion, we labeled RNAP as β′-mEos2 expressed from the chromosome (Bakshi et al., 2013). Single-molecule diffusive trajectories of 1-s duration cleanly distinguish two states of motion of RNAP. In **Figure 5**, compare the compact purple trajectory with the extended yellow one. Specifically bound copies jiggle in place (sub-diffusion), much like DNA foci, while copies searching for transcription initiation sites undergo apparently free diffusion (a combination of 3D hopping and non-specific binding) within the nucleoids. For growth in EZRDM at 37°C, about 50% of RNAP copies are evidently specifically bound to DNA (including all stages of transcription). According to classic estimates (Dennis et al., 2004), about 1/2 to 2/3 of this 50% should be transcribing stable RNA (rRNA and tRNA); the remainder should be transcribing protein genes. In rapidly growing cells, both widefield (Jin and Cabrera, 2006; Bratton et al., 2011) and super-resolution (Endesfelder et al., 2013) fluorescence studies find highly concentrated clusters of RNAP copies. These “transcription foci” presumably comprise RNAP copies engaged in transcription of *rrn* operons.

**FIGURE 6:**
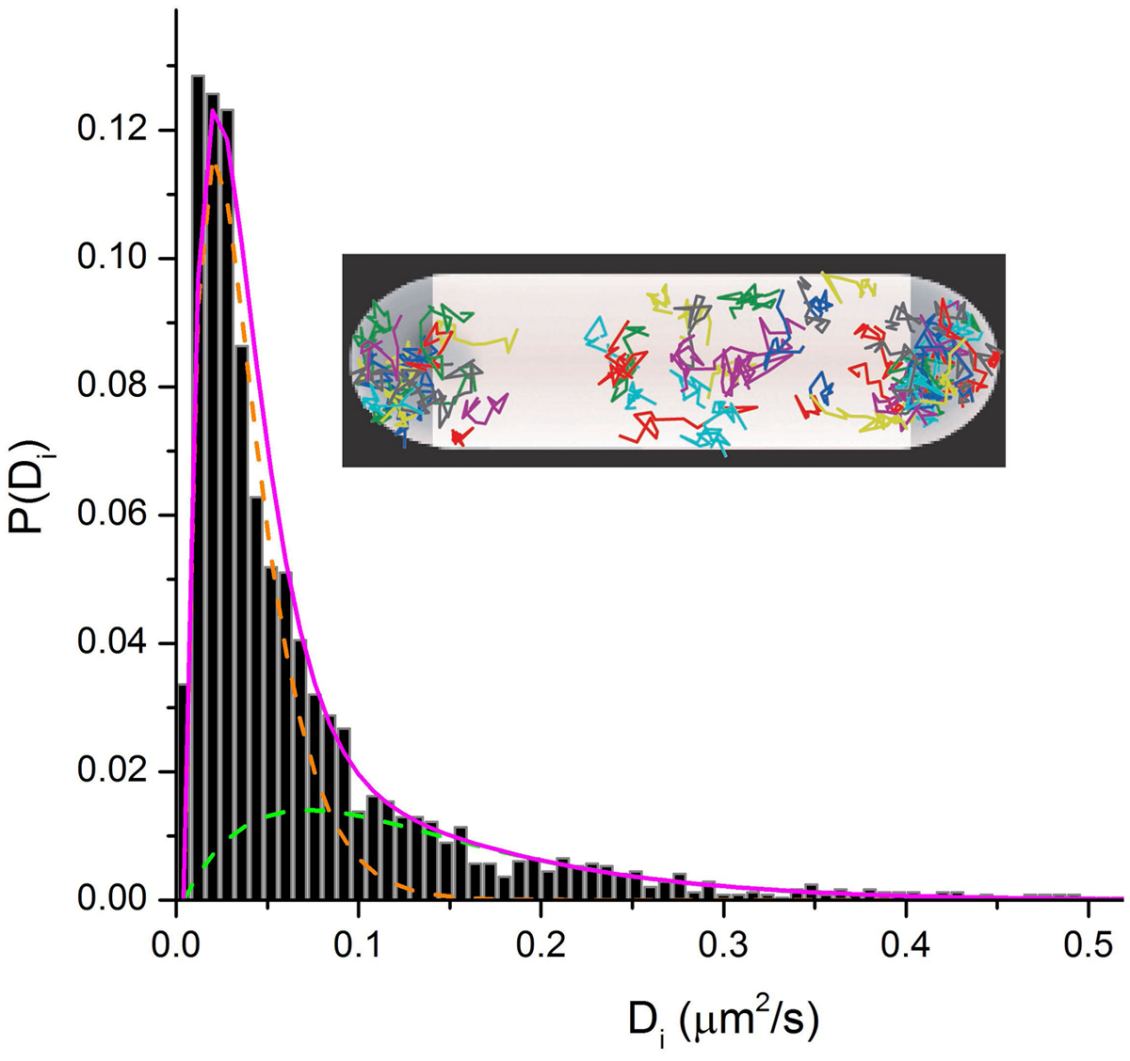
**Inset:** Single-ribosome diffusive trajectories from a live *E. coli* cell growing in EZRDM at 30°C. Labeling is S2-YFP. **Main figure:** Distribution of estimated single-ribosome diffusion coefficients *D_i_*. S2-mEos2 labeling. Two sub-populations are evident. Model sub-distributions are shown as green and orange dashed lines. Magenta line is their sum.

In the ribosome imaging studies, we found that some 85–90% of the 30S-YFP copies lie in the “ribosome-rich regions” (Bakshi et al., 2012). This suggests that only ∼10–15% of translation is co-transcriptional. The diffusive behavior of single 30S-mEos2 copies (**Figure 6**) reveals two distinguishable sub-populations. This enabled us to roughly divide the 30S behavior into 20–30% free 30S with diffusion coefficient *D_30S_* ∼ 0.14 μm^2^ s^-1^ and 80–70% 70S-polysomes with *D_70S-poly_* ∼ 0.02 μm^2^ s^-1^. The latter value describes the diffusive motion of mRNA decorated by a variable number of 70S-ribosomes. For comparison, the timescale of transcription of mRNA for a typical protein is ∼20 s and the degradation time for messages is ∼5 min (Bernstein et al., 2002). These data in *E. coli* are consistent with the view that most transcription of protein genes is co-translational. However, mRNA copies spend most of their lifetime separated from RNAP and DNA. After completion of transcription, *D_70S-poly_* is large enough to enable free mRNA decorated with translating 70S ribosomes to diffuse to a ribosome-rich region in ∼1 s. There each message can be translated repeatedly before degradation.

**FIGURE 7:**
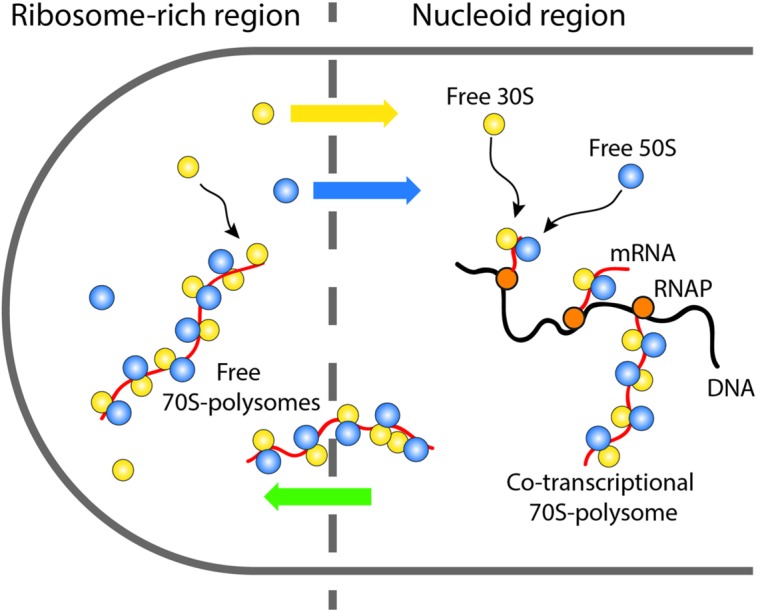
**Schematic showing the suggested circulation of ribosomal subunits into and out of the nucleoids and the ribosome-rich regions**.

After complete synthesis of a protein in a ribosome-rich region, the newly free 30S and 50S subunits may engage in repeated rounds of translation in the ribosome-rich region, or “escape” back to the nucleoid region where they can re-initiate co-transcriptional translation (**Figure 7**). Accordingly, the Elf lab recently showed that the more rapidly diffusing 30S and 50S subunits penetrate the nucleoids while the slower 70S-polysomes are largely excluded (Sanamrad et al., 2014). This picture implies a circulation of ribosomal subunits from the nucleoids (where co-transcriptional translation occurs) to the ribosome-rich regions (where most protein synthesis occurs) and back again.

In fast growth conditions, concentration of 70S-polysomes in ribosome-rich regions may enhance the rate of protein synthesis by shortening the search time for translation initiation sites by newly freed 30S and 50S subunits that have just completed synthesis of a protein. Such spatial separation of transcription from most translation in *E. coli* is somewhat reminiscent of eukaryotic cells. However, it occurs without compartmentalization of DNA within a nuclear membrane. The Monte Carlo simulations described below suggest that the underlying segregation mechanism may be primarily physical in nature.

## What Driving Forces Induce Nucleoid-Ribosome Mixing or Segregation?

In spite of the 1.5 mm contour length of an *E. coli* chromosome, the nucleoids do not fill the entire volume of the 3–4 μm long, 1 μm diameter cytoplasm (Pettijohn, 1982; Robinow and Kellenberger, 1994). The irregular shape of the *E. coli* nucleoids may be governed by the ring topology of DNA and spatial confinement effects on the ring polymer (Jung et al., 2012; Fisher et al., 2013; Youngren et al., 2014). In *C. crescentus*, chromosome conformation capture (3C) data suggest the nucleoids adopt a “bottle brush” geometry with plectonemes radiating outward from a central spine (Le et al., 2013). Less detail is presently available for the *E. coli* chromosome (Cagliero et al., 2013). Our focus here is on the coarse spatial extent of the nucleoids under different conditions, not the finer details.

### Long-Term Effects of Chloramphenicol and Rifampicin on Nucleoid-Ribosome Morphology

**FIGURE 8:**
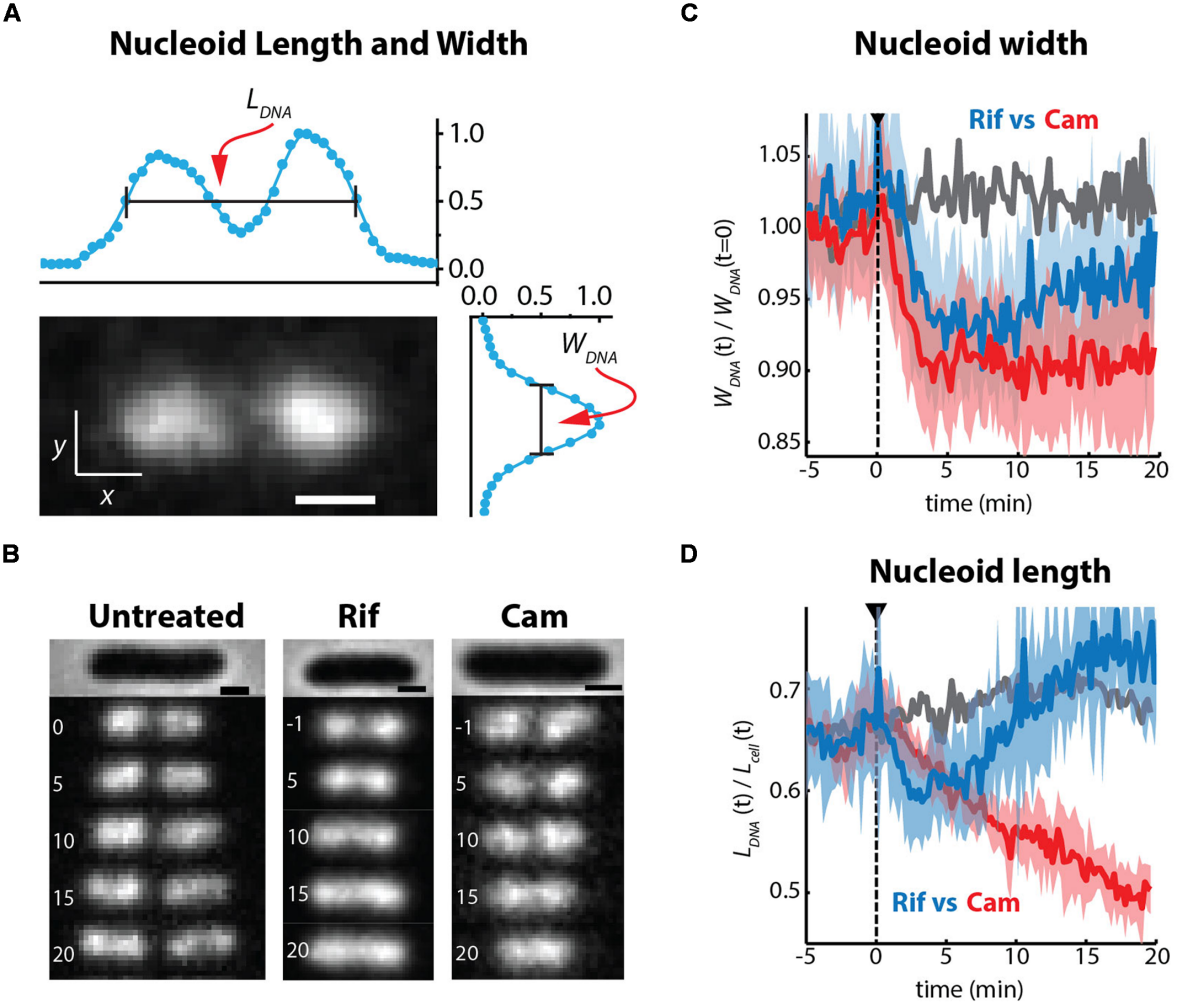
**(A)** SYTOX orange-stained image of chromosomal DNA in a live *E. coli* cell growing in EZRDM at 30°C. The nucleoid spatial extent is characterized by length *L_DNA_* and width *W_DNA_* measured as the full width at half-maximum height (FWHM) of intensity distributions projected onto the *x* and *y* axes. **(B)** Time-lapse sequences of images of nucleoids stained by SYTOX Orange. Times in minutes, scale bars are 1 mm. Untreated cells, Rif-treated cells, and Cam-treated cells as indicated. **(C)** Quantitative nucleoid width *W_DNA_* vs. time. *Gray:* mean behavior of a set of untreated cells. *Blue:* behavior of Rif-treated cells. *Red:* behavior of Cam-treated cells. For blue and red, heavy lines are averages of traces from many cells; shaded regions show the envelope of single-cell results that were averaged. Dashed line shows time of initiation of flow of drug. **(D)** Same as **(C)**, but with relative nucleoid length plotted as *L_DNA_*/*L_cell_*. Adapted from Bakshi et al. (2014a).

It has long been known that transcription- and translation-halting drugs strongly affect nucleoid morphology. Typical imaging experiments have compared nucleoid morphology before and 20–30 min after drug treatment, usually using DAPI as the DNA stain. We used the non-perturbative DNA stain SYTOX Orange (Bakshi et al., 2014b) to study *time-dependent, quantitative* effects of Rif and Cam on nucleoid length and width in live cells over 20 min (Bakshi et al., 2014a). To describe the overall spatial distribution of the chromosomal DNA vs. time, we defined two parameters measured from the SYTOX Orange fluorescence intensity distributions projected along the *x*- and *y*-axes (**Figure 8A**). The axial distribution was characterized by the overall length *L_DNA_*, measured as the “outside” full-width at half-maximum height (FWHM) of the projected intensity distribution along *x*. The width *W_DNA_*, a rough measure of the mean nucleoid diameter, was defined as the FWHM of the projection of intensity along the transverse coordinate *y*. We believe this definition of nucleoid length and width is more quantitative than the “relative nucleoid size” used in other work (Jin et al., 2013). We also measured single-ribosome diffusive motion vs. time after drug treatment, using the 30S-mEos2 labeling scheme (Bakshi et al., 2014a). This helps distinguish 70S-polysomes from free 30S subunits after drug treatment.

On the 10–20 min timescale, the nucleoids of Cam-treated cells have become much more compact axially than normally growing cells, while the nucleoids of Rif-treated cells have expanded in both dimensions (**Figures 8B,C**). Similar long-term drug effects were observed in earlier work (Cabrera and Jin, 2006; Zimmerman, 2006). Ribosomes are evidently maintained as 70S-polysomes on a 20-min timescale after Cam treatment, as inferred from the distribution of ribosome diffusion coefficients (Bakshi et al., 2014a). We further suggest that the long-term nucleoid expansion induced by Rif is due to slow degradation of existing mRNA, after transcription initiation is halted by the drug. Imaging of the putative mRNA stain SYTO RNASelect vs. time is consistent with this suggestion. As mRNA is degraded, 70S-polysomes that dissociate to 30S and 50S subunits after completion of translation find fewer and fewer translation initiation sites. The resulting free subunits diffuse much more rapidly than 70S-polysomes. Unlike 70S-polysomes, the free subunits mix thoroughly with the nucleoids.

### Nucleoid-Ribosome Mixing Hypothesis

**FIGURE 9:**
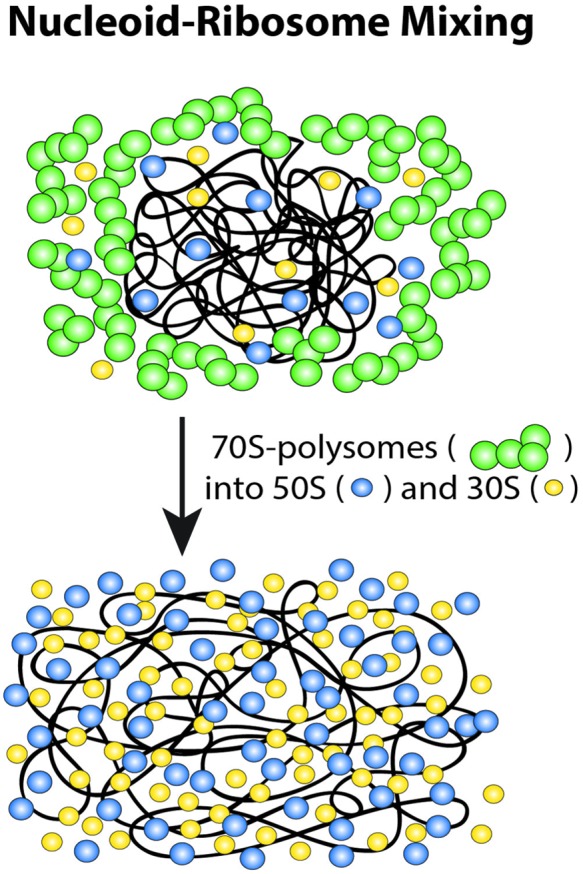
**Schematic of ribosome-nucleoid mixing hypothesis.** 70S-polysomes and DNA strongly avoid each other, while free 30S and 50S subunits readily penetrate into the nucleoids. Adapted from Bakshi et al. (2014a).

These long-term drug effects have motivated a new nucleoid-ribosome mixing hypothesis (**Figure 9**; Bakshi et al., 2014a). We view the ribosomes and the chromosomal DNA as a *composite biochemical–biophysical system*. Cell physiology and drug treatments dictate the partitioning of ribosomal components between 70S-polysomes and free 30S and 50S subunits. Overall nucleoid spatial extent is then governed by the tendency for nucleoids to segregate from 70S-polysomes and to mix with free 30S and 50S subunits. The Monte Carlo modeling (Mondal et al., 2011; Bakshi et al., 2014a) described next suggests that the underlying driving forces for mixing or segregation are *excluded volume effects* combined with *maximal total entropy* of the composite DNA-ribosome system.

In this view, the drug studies reveal three “states” of the nucleoid-ribosome system. For normally growing, untreated cells, transertion expands the nucleoids, imposing a state of *intermediate DNA density* that prevents 70S polysomes and the chromosomal DNA from mixing while permitting 30S and 50S subunits to penetrate the nucleoids. Transertion may thus be essential for the initiation and maintenance of co-transcriptional translation of nascent mRNA in the dense regions of the nucleoids. Cam freezes ribosomal subunits as 70S-polysomes. Completion of transcription events will break the transertion chains and the 70S-polysomes will then be unable to make new chains. Over 20 min, the chromosomal DNA relaxes to a *fully condensed state* that excludes both 70S-polysomes and 30S and 50S subunits. The fraction of free 30S and 50S subunits becomes even smaller than in normal growth, enabling very strong compaction of the nucleoids. We suggest that this represents the relaxed volume of the nucleoids in the absence of the expanding force of transertion and in the near absence of free ribosomal subunits. Rif treatment also breaks the transertion links on the same timescale as Cam, the time over which transcription events are completed. Again, new transertion chains are prevented from forming. This leads to similar short-term contraction (see below). DNA mixing with 30S and 50S ribosomal subunits eventually occurs on the longer, 10–20 min timescale of mRNA degradation. This leads to the third, *fully expanded* state of the nucleoids, in which few 70S-polysomes exist and DNA and the 30S and 50S ribosomal subunits mix extensively. However, the nucleoids continue to avoid the cylindrical walls and especially the endcaps.

### Simple Physical Model of Ribosomes and Nucleoids

**FIGURE 10:**
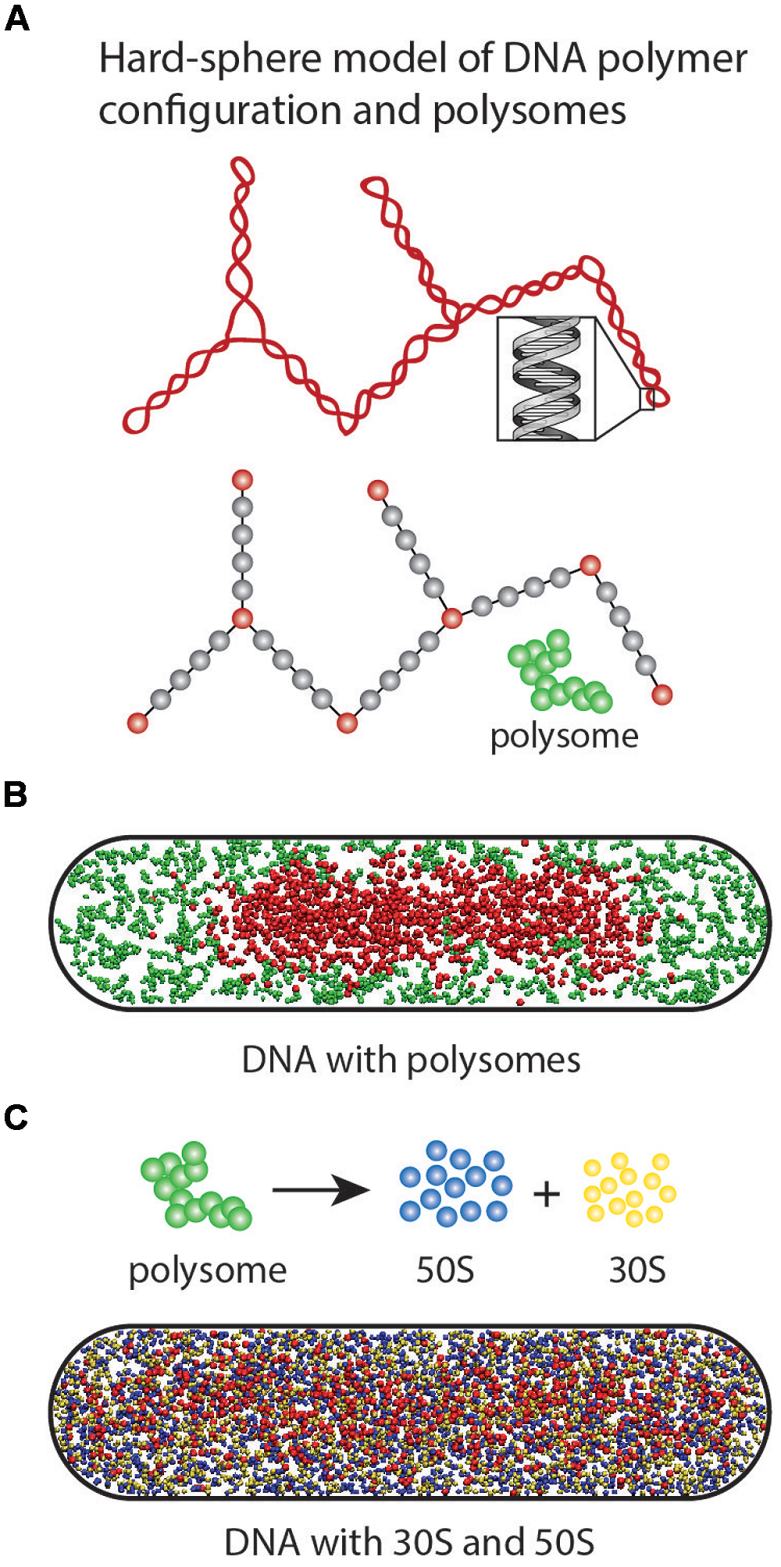
**(A)** Hyper-branched polymer bead model of plectonemic DNA and 70S-polysomes. The red DNA beads exclude each other; the gray beads are invisible to the DNA beads but act as volume appropriately excluded to the polysomes. Polysomes are represented as freely jointed chains of spheres of appropriate size. **(B)** In Monte Carlo simulations, 70S-polysomes and DNA strongly avoid each other. **(C)** When the 70S-polysomes are dissociated into 50S and 30S monomers, the simulations show strong mixing and nucleoid expansion. Adapted from Bakshi et al. (2014a).

In Mondal et al. (2011), we developed a simple physical model of plectonemic DNA and 70S-polysomes confined in a spherocylinder (as pictured in **Figure 10A**). DNA was modeled as a hyperbranched polymer (hard spheres and connecting rods). Based on estimates at the time (Bremer and Dennis, 1996), the model placed two chromosome equivalents of plectonemic DNA (comprising 7000 plectoneme rods) plus 20,000 70S particles organized as freely jointed 70S-polysome 13-mers into a spherocylinder (350 nm radius, 3.0 μm length). DNA–DNA, polysome–DNA, and polysome–polysome excluded volume effects were modeled realistically. There are no attractive interactions between particles. The free energy of the composite system was minimized using Monte Carlo methods. This minimalist, coarse-grained model does not include the effects of transertion, nor does it attempt to describe the level of geometric detail revealed by recent chromosome conformational capture data (Cagliero et al., 2013; Le et al., 2013; Le and Laub, 2014). Instead, it seeks to understand the effects of excluded volume and entropy on the overall spatial distributions of DNA and ribosomes.

Translational entropy is the entropy of movement in space. It increases with the number of free particles and with the volume available to the particles. DNA conformational entropy is determined by the number of conformational states available to the DNA polymer. *In the model, these entropic plus excluded volume effects lead to strong segregation of DNA from 70S-polysomes* (**Figure 10B**). The model DNA polymer, comprising beads connected by rods, avoids the walls and does not fill the cytoplasm. This is because placing a bead near a wall would eliminate many potential conformations of the polymer and hence decrease conformational entropy.

To help understand why Rif-induced dissociation of 70S-polysomes into 30S and 50S subunits enables mixing of free subunits with the DNA and nucleoid expansion, we have recently performed a new set of simulations based on the same simplified model of DNA (**Figure 10C**; Bakshi et al., 2014a). The 20,000 70S ribosomes engaged as 1538 polysome 13-mers were converted to 20,000 30S spheres and 20,000 50S spheres. Excluded volume effects were adjusted appropriately. *The free 30S and 50S subunits mix with the DNA polymer*. In addition, the nucleoid becomes more expanded, while continuing to avoid the confining walls. Both features are reminiscent of experimental results (Bakshi et al., 2014a; Sanamrad et al., 2014).

The model suggests that the primary driving force for mixing of chromosomal DNA with ribosomal subunits after Rif treatment is *increased translational entropy*. Each 70S-polysome 13-mer has become 26 independent subunits, each “demanding” its own translational entropy. We suggest that the nucleoid expands to provide the 30S and 50S subunits with access to more volume in which to move.

In real cells, the nucleoids adopt their most compact form after Cam treatment, which preserves most ribosomal subunits as 70S-polysomes. Importantly, in this highly compacted form the nucleoids exclude not only 70S-polysomes, but also free 30S subunits (Sanamrad et al., 2014), LacI (Kuhlman and Cox, 2012), and the Kaede tetramer (Bakshi et al., 2011). Accordingly, we believe that it is *transertion* that expands the nucleoids sufficiently to enable 30S and 50S subunits to penetrate and initiate co-transcriptional translation.

Co-transcriptional translation in turn is biologically important for preventing premature degradation of mRNA by endonucleases, termination of transcription by Rho, and excessive backtracking by RNAP. *Thus transertion, whose very existence has long been debated, may be essential to optimal cell growth*.

In our view, the overall physiological state of the cell (slow growth, fast growth, stationary phase, stress response, etc.) determines the number of ribosomal subunits and their partitioning between 70S-polysomes and 30S and 50S subunits. The Monte Carlo modeling then suggests that at least in rapid growth, the corresponding coarse nucleoid morphology is dictated by the tendency of the combined nucleoid-ribosome *system* to maximize total conformational and translational entropy. The modeling further suggests that in the presence of free 30S and 50S subunits, the tendency to maximize translational entropy of ribosomal subunits provides a second expanding force on the nucleoids, in addition to transertion. This tendency is most evident after treatment with the transcription-halting drug Rif. It is less evident, but may still be a significant effect, in normal growth when 30S and 50S subunits are minority ribosomal species. Our model of the factors controlling the overall DNA spatial distribution might be called “translation-centric,” because it emphasizes the importance of the DNA-ribosome system.

It is difficult to compare our model of DNA-ribosome interactions with other physical models of DNA confinement and compaction. The two most recent models treat the DNA polymer as a freely jointed chain of beads (Pelletier et al., 2012; Shendruk et al., 2015), whereas we represent DNA as plectonemes modeled as a hyper-branched chain of sticks connecting beads. The other models include small-protein crowders but not ribosomes, whereas we include ribosomes explicitly and neglect small-protein crowders. With its 7000 connecting rods, our hyper-branched chain has many more internal degrees of freedom than a freely jointed chain of several 100 beads, and so may have a greater tendency to avoid walls. We anticipate more detailed physical models in the future, perhaps including crowders of all sizes, the effects of transertion, and new information about specific DNA conformations from chromosome-capture experiments (Cagliero et al., 2013; Le et al., 2013).

## New Evidence for Transertion

### Background

Transertion is viewed as a dynamic process in which numerous membrane-DNA linkages are constantly forming and breaking during normal transcription and translation events (Norris and Madsen, 1995). The transertion hypothesis implies DNA-RNAP-mRNA-ribosome-polypeptide-membrane “transertion chains” that tether the chromosomal DNA to the cytoplasmic membrane and provide a radially expanding force on the nucleoids (**Figure 1**). The primary evidence for transertion had long been the dramatic contraction of the nucleoids after treatment with drugs that halt translation (e.g., chloramphenicol; van Helvoort et al., 1996; Zimmerman, 2002). Such treatment should break the transertion chains on the timescale of completion of transcription events (∼20 s). However, transertion chains should also be broken by drugs such as rifampicin (“Rif”), which prevents transcription initiation. Yet cells observed 20–30 min after Rif treatment showed nucleoid *expansion*, in contradiction of the transertion hypothesis (Jin and Cabrera, 2006).

Recent additional support for transertion came from the Goulian lab. Libby et al. (2012), they discovered a net outward migration of genes encoding cytoplasmic membrane proteins within minutes of induction of transcription. They measured the 2D spatial distribution of fluorescent markers of genes encoding two soluble proteins (*mcherry*, coding the fluorescent protein mCherry; and *aadA*, spectinomycin adenylyltransferase) and of genes encoding two membrane proteins (*lacY*, lactose permease; and *tetA*, the tetracycline eﬄux pump). For the two membrane-protein genes (but not for the soluble protein genes), induction caused a substantial outward shift in the distribution (toward the membrane), consistent with the transertion hypothesis. The shift occurred within 1–3 min of gene induction.

### Short-Time Imaging of Drug Effects on Nucleoid Morphology

Our recent time-resolved drug studies have resolved the Rif dilemma (Bakshi et al., 2014a). The non-perturbative DNA stain SYTOX Orange allows monitoring of nucleoid morphology in growing cells using widefield time-lapse microscopy. We are able follow the size and shape of the nucleoids at 10 s intervals over 25 min after cells are treated with drugs. *On the 0–5 min timescale, both Cam and Rif shrink both the length and width of the nucleoids* (**Figures 8B–D**). This is consistent with the transertion hypothesis. Both Cam and Rif should cause radial contraction of the nucleoids on the timescale of completion of transcription events within the transertion chains. On the 10–20 min timescale following Rif treatment, the nucleoids of the Rif-treated cells expand axially, becoming longer than in the unperturbed state. As described above, we believe the expansion is due to converstion of 70S-polysomes to 30S and 50S subunits, which freely mix with the nucleoids. However, the nucleoid width remains smaller than that of the unperturbed state, consistent with wall avoidance by the DNA polymer, which is no longer tethered to the plasma membrane.

## A “Transcription-Centric” Model of *E. coli* Organization

In a separate chapter of this compendium, Ding Jin presents a very different view of the factors governing the overall DNA spatial distribution. The model from the Jin lab might be called “transcription-centric.” Jin et al. (2013) labeled RNAP using a GFP tag on the β′ subunit and imaged fixed *E. coli* cells harvested from rapidly growing cultures. They discovered that each cell exhibits several bright puncta of RNAP-GFP intensity, which they dubbed “transcription foci” (Cabrera et al., 2009). Similar foci have been observed in a recent super-resolution study (Endesfelder et al., 2013). The Jin lab studied the presence or absence of transcription foci as well as the overall nucleoid spatial extent in a wide variety of growth and stress conditions (Jin et al., 2013). Transcription foci dissipate rapidly when fast-growing cells are starved or treated with Rif, among other conditions. The foci are not evident in slowly growing cells.

The transcription foci are very likely concentrated centers of transcription of *rrn* operons, the predominant type of transcription in rapidly growing cells. The seven *rrn* operons are distributed broadly within the half of the chromosome closest to *oriC*. Rapidly growing cells containing perhaps four genome equivalents of DNA can easily harbor ∼40 *rrn* operons, a number that far exceeds the number of transcription foci observed. This strongly suggests *clustering* of *rrn* operons into hubs of rRNA transcription, perhaps analogous to the nucleolus of eukaryotic cells. The cause of such clustering is unknown. Cook and co-workers developed a statistical model that suggested that a strong depletion-attraction force would arise between two *rrn* operons heavily decorated with RNAP copies (Marenduzzo et al., 2006).

Across a variety of growth conditions, mutant strains, and drug treatments, Jin et al. (2013) observed a strong, positive *correlation* between the presence of transcription foci and the occurrence of relatively compact states of the nucleoids. This led to the suggestion that binding of a large fraction of RNAP copies within transcription foci somehow *causes* the nucleoid to compact.

The most recent concept involves the redistribution of RNAP copies that occurs in conditions that “dissolve” transcription foci. Imaging evidence indicates that the transcription foci tend to lie at or near the nucleoid periphery (Jin et al., 2013). In rapidly growing cells, RNAP may thus be depleted from the bulk of the nucleoids. When the RNAP previously concentrated in peripheral *rrn* clusters are dispersed by Rif treatment or by inducing the stringent response, they bind specifically and non-specifically to numerous sites within the bulk of the DNA. The suggestion is that this expands the nucleoids. However, such expansion cannot be due to insertion of the extra volume of RNAPs into the bulk of the nucleoids. The nucleoids occupy roughly half of the total cytoplasmic volume, or ∼2 μm^3^ in rapid growth. The RNAP copy number per cell is ∼5000 (Bakshi et al., 2012). Even if we place all the RNAP copies within the nucleoids, their total volume is only ∼0.003 μm^3^. Insertion of RNAP copies into the bulk of the nucleoids in and of itself cannot induce anything like the observed volume expansion induced by Rif. Similarly, the presence in the nucleoid of 10–15% of the ribosomes has only a minor effect on overall nucleoid volume (Bakshi et al., 2012). The total volume of 7,500 70S ribosomes is only ∼0.03 μm^3^.

Instead, we believe it is the translational entropy of free ribosomal subunits that drives the expansion of the nucleoid (Bakshi et al., 2014a). The fraction of ribosomes involved in active translation as 70S polysomes decreases upon Rif treatment or induction of the stringent response. The increased contribution of translational entropy from the increased fraction of free, non-translating ribosomal subunits causes the nucleoid to expand, making the entire the cytoplasm accessible to the 30S and 50S subunits (**Figures 9** and **10**).

It might be suggested that the gathering of multiple *rrn* operons into a single *rrn* cluster, whatever the underlying cause, constrains the entire nucleoid to be more compact. A critical test of this suggestion would use dual-color fluorescence reporter–operator systems (FROS) to label two different *rrn* operons and test whether *rrn* clusters persist or disperse in slow growth. If they disperse and the nucleoids expand, the idea may have merit. At present, a counter-argument is that the linear contour of one genome measures 1.5 mm in length. Whatever causes *rrn* operons to cluster, it seems likely to us that there is plenty of “slack” in the overall chromosome to accommodate clustering of distant operons without compacting the overall nucleoid morphology. Additional study of the confined DNA polymer model, including pinning together of distant beads to each other, may be informative here.

Also relevant is a recent study of DAPI-stained nucleoid morphology in different growth conditions (Kuhlman and Cox, 2012). From a quantitative comparison of the integrated curvature of 2D images of the nucleoids, they inferred that the nucleoids are substantially *more condensed* (have higher density) in slowly growing cells than in rapidly growing cells. Since transcription foci do not occur in slowly growing cells, this would appear to oppose the trend predicted by the transcription-centric model of Jin et al. (2013).

We plan to extend our ribosome, RNAP, and nucleoid imaging and tracking studies to very slow growth conditions to test the nucleoid-ribosome mixing hypothesis in a very different physiological context.

## Does mRNA Co-localize with Ribosomes or with the Gene from Which it was Transcribed?

According to our model of *E. coli* spatial organization, in rapid growth most translation is physically separated from most transcription. The exception is the small fraction of co-transcriptional translation (perhaps 10–15%), which should co-localize with the chromosomal DNA. If this picture is essentially correct, then the spatial distribution of total mRNA should mimic that of the ribosomes more closely than that of the chromosomal DNA.

As reviewed by Amster-Choder and co-workers (Buskila et al., 2014), there are two primary methods for labeling specific mRNAs: fluorescence *in situ* hybridization (“FISH,” which requires fixation and permeabilization of the cells) and tagging of mRNA with a sequence to which a fluorescently labeled protein such as MS2-GFP binds specifically (which can be carried out on live cells). That review describes the current evidence on mRNA localization. The resulting picture is complicated and far from complete. It is possible that the picture of strong ribosome-nucleoid segregation and rapid diffusion of mRNA-70S polysomes away from their point of origin after completion of transcription holds only for rapidly growing cells.

### Results in Rapidly Growing *E. coli* and *B. subtilis*

Early on, Golding and Cox (2004) used the MS2 scheme to label a very large, artificial mRNA transcribed from a plasmid. The resulting MS2-coated mRNA copies have mass of several MDa, comparable to a ribosome. They localized near the cell poles, much like ribosomes. The diffusive and localization properties of such large objects are not closely related to those of normal gene transcripts.

Amster-Choder and co-workers labeled specific *E. coli* genes in live cells under fast growth conditions using the MS2-GFP procedure (Nevo-Dinur et al., 2011). They found that the mRNA coding for the membrane proteins *lacY* and *bglF* was *localized at the membrane*. This behavior persisted even after treatment with translation-halting drugs such as Cam. The interpretation was that the mRNA itself contains information that targets the transcript to the location where the protein will ultimately be used, analogous to what occurs in eukaryotic cells. However, if the lead ribosome (i.e., the one connected to the RNA polymerase) is indeed necessary to prevent premature intrinsic or Rho-dependent termination of transcription (Burmann et al., 2010), then Cam may prevent efficient transcription as well. We suggest that this result might be interpreted as further evidence of transertion chains. The ribosome-mRNA-membrane linkages would persist after Cam treatment, so the localization of mRNA at the membrane would also persist.

In contrast, the mRNAs coding for the cytoplasmic proteins *cat* and *bglB* were distributed throughout the cytoplasm in a pattern that was described as helical (Nevo-Dinur et al., 2011). However, to our eyes the *cat* and *bglB* mRNA distributions look like the strongly segregated ribosomal spatial distributions in fast growth conditions (**Figure 4**).

We recently used the fluorescent stain SYTO RNASelect to monitor the degradation of total mRNA following Rif treatment (Bakshi et al., 2014a). SYTO RNASelect purportedly stains RNA in preference to DNA. For normally growing cells in EZRDM at 30°C, the resulting images look qualitatively similar to widefield images of ribosomes labeled by S2-YFP in the same growth conditions (**Figure 3**; Bakshi et al., 2012). The *caveat* here is the possibility that SYTO RNASelect is actually staining ribosomal RNA, not mRNA. However, most rRNA is buried in the ribosome interior and not accessible to the stain. In addition, the SYTO RNASelect signal decayed on a 10-min timescale after Rif treatment, consistent with the timescale of degradation of mRNA. Ribosomes are still present after the decay of SYTO RNASelect fluorescence.

Allowing for some re-interpretation of the Amster-Choder results, the available data on mRNA distributions from live, rapidly growing *E. coli* cells seems quite consistent with our overall picture of strong ribosome-DNA spatial segregation in rapidly growing cells.

Rapidly growing *B. subtilis* cells also exhibit strong nucleoid-ribosome segregation (Lewis et al., 2000). The first two-color widefield studies of cells with DNA stained and ribosomes labeled showed two or three ribosome-rich regions with interleaved regions of concentrated DNA. We know of no data on mRNA spatial distribution in *B. subtilis*, but we expect the distribution would closely mimic the ribosome distribution.

### Results in Slowly Growing *C. crescentus* and *E. coli*

Using FISH in the slowly growing species *C. crescentus*, the Jacobs-Wagner lab found that six different mRNA messages formed images comprising one or a few *puncta* (Llopis et al., 2010). Remarkably, each of these puncta co-localized with the corresponding gene (also detected by FISH). In addition, the ribosomes and DNA mix extensively in *C. crescentus*. The mixing means that there is no contradiction between retention of mRNA near the site of its transcription and repeated translation of the same message. In slowly growing *E. coli*, similar mRNA and gene co-localization was observed for *lacZ*. This suggests that in slow growth conditions, mRNA diffusion away from the gene from which it was transcribed is very slow, in contrast to our picture of facile mRNA escape from the nucleoids in rapid growth conditions.

In a similar vein, Kuhlman and Cox (2012) used DAPI staining, “FROS” (a fluorescent reporter–operator system), FISH, and Venus labeling in *E. coli* to measure the spatial distributions (averaged over many cells) of the overall nucleoid, the *lacI* gene, its *lacI* mRNA product, and its LacI-Venus protein product. In slow growth, they varied the position of the *lacI* gene, either on an extrachromosomal plasmid, near *oriC*, or near *ter*. The gene and mRNA distributions are non-uniform and quite different in the three cases, exhibiting two, one, and three axial peaks, respectively. In all three cases, the mRNA distribution closely mimicked the gene distribution, again suggesting that the mRNA is not readily diffusing away from the location where it was transcribed. It is perhaps worth noting that the three different *lacI* gene and mRNA spatial distributions in slow growth conditions all have strong peaks in locations that would match our three ribosome-rich regions in rapidly growing cells. However, in rapidly growing *E. coli*, Kuhlman and Cox (2012) found that the *lacI* mRNA spatial distributions from FISH mimic the overall nucleoid distributions, again suggesting that the mRNA does not readily escape the nucleoids. That is, the *lacI* mRNA does not form a pattern mimicking the *ribosome* spatial distributions of **Figures 3** and **4**.

In slowly growing *E. coli* the evidence for two different genes (*lac Z* and *lacI*) indicates that the corresponding mRNA message remains in the vicinity of the gene itself rather than diffusing away (Kuhlman and Cox, 2012). It is possible that the behavior of *lacZ* and *lacI* mRNA in slowly growing cells is somehow not representative of the predominant mRNA behavior. It is also possible that strong DNA-ribosome segregation only occurs in rapidly growing cells. However, facile DNA-ribosome mixing in slow growth would seem contrary to the Kuhlman and Cox (2012) inference of higher DNA density in slowly growing cells than in rapidly growing cells. According to our Monte Carlo modeling, higher DNA density in slowly growing cells would exclude 70S-polysomes to a greater extent, assuming that they can escape the nucleoid interior in the first place.

At present, the mRNA data on slowly growing cells, both *E. coli* and *C. crescentus*, stand in opposition to a picture of strong DNA-ribosome segregation and diffusion of free mRNA to ribosome-rich regions where the bulk of translation occurs. We have not carried out detailed super-resolution studies of ribosomes in slowly growing *E. coli*. We plan to repeat the spatial distribution and diffusion studies to measure the degree of nucleoid-ribosome segregation and the diffusive properties of the ribosomes. Studies of the SYTO RNASelect staining pattern in slow growth are also of interest.

Returning to rapidly growing *E. coli* cells, it seems highly implausible to us that the nucleoids and ribosomes would be strongly segregated while the *overall* mRNA spatial distribution would mimic that of the chromosomal DNA. If that were the case, then what are the ∼80% of ribosomes doing while they are so far from the majority of the mRNA that they must translate? The situation could be quite different in slowly growing *E. coli*. Additional work is needed.

## Summary

We have presented a comprehensive model whose goal is to explain the coarse spatial organization of the *E. coli* transcription and translation machinery in rapid growth conditions with and without the influence of transcription- or translation-halting drugs. This builds on a great deal of earlier work from other labs (Odijk, 1998; Pelletier et al., 2012). Our model takes account of both the biochemical state of the cell (fraction of ribosomes engaged in translation, presence or absence of transertion chains) and the important underlying physical effects of excluded volume and conformational and translational entropy. By treating the ribosomes and DNA as a coupled biochemical–biophysical *system*, the model explains a wide variety of experimental results.

A wise cell biologist once complained that the trouble with physics-based hypotheses attempting to explain aspects of cellular behavior is that they can never be critically tested. There are no appropriate negative controls—you cannot turn the physics off. Our response would be that neither should the physics be neglected in our thinking. Physical models that make experimentally testable predictions have real value in cell biology. Our physical model can explain why in normal, rapid growth conditions 70S-polysomes and the nucleoids segregate, while free 30S and 50S subunits mix with the nucleoids. The model is oversimplified to be sure, but we believe it captures essential interactions that must not be ignored. From the time-dependent drug studies, we further infer that transertion maintains the nucleoids in a sufficiently expanded state to enable recycling of 30S and 50S subunits between ribosome-rich regions and the nucleoid interior, where they initiate co-transcriptional translation. This in turn protects the nascent mRNA and prevents undesirable backtracking of RNA polymerase.

These concepts suggest how the strongly coupled transcription-translation-transertion *system* may enhance the maximum growth rate of *E. coli*. Strong segregation of DNA and RNAP from 70S-polysomes in rapid growth may significantly decrease the search times of RNAP for transcription initiation sites and of free 30S and 50S ribosomal subunits for translation initiation sites. Future work will provide additional quantitative detail in rapid growth and seek a better understanding of the situation in slowly growing cells.

## Conflict of Interest Statement

The authors declare that the research was conducted in the absence of any commercial or financial relationships that could be construed as a potential conflict of interest.
